# Non-compliance with COVID-19 Health Recommendations: Five- and Ten-Month Effects on Mental Health and Academic Self-efficacy Among University Students in Sweden

**DOI:** 10.1007/s12529-024-10343-w

**Published:** 2024-12-30

**Authors:** Claes Andersson, Anne H. Berman, Petra Lindfors, Marcus Bendtsen

**Affiliations:** 1 Department of Criminology, Malmö University, 205 02 Malmö, Sweden; 2 Department of Psychology, Uppsala University, Uppsala, Sweden; 3 Department of Psychology, Stockholm University, Stockholm, Sweden; 4 Department of Health, Medicine and Caring Sciences, Linköping University, Linköping, Sweden

**Keywords:** Non-compliance, COVID-19, Public health recommendations, Mental health, Academic self-efficacy, Longitudinal study

## Abstract

**Background:**

Addressing the effects of non-compliance with health-related recommendations in pandemics is needed for informed decision-making. This longitudinal study investigated the effects of non-compliance on mental health and academic self-efficacy among university students in Sweden.

**Methods:**

Baseline assessments were conducted in May 2020, with follow-ups after 5 and 10 months. Students (*n* = 3123) from 19 universities completed online questionnaires covering compliance, mental health, and academic self-efficacy. Effects of non-compliance were estimated using causal inference and multilevel multinomial regression.

**Results:**

Non-compliant students constituted a minority, but their proportion increased over time. Regarding mental health and academic self-efficacy, few differences were observed between compliant and non-compliant students. When differences were identified, non-compliant students experienced fewer negative effects on mental health and academic self-efficacy than compliant students.

**Conclusion:**

The findings may suggest that non-compliance may have involved a trade-off between increased individual freedom and mitigating negative outcomes. Addressing the research gap on non-compliance effects is crucial for informed decision-making and promoting the common good. This may guide strategies balancing individual autonomy and collective well-being during future pandemics.

**Pre-registration:**

Center for Open Science (OSF), https://accounts.osf.io/login?service=https://osf.io/37dhm/.

**Supplementary Information:**

The online version contains supplementary material available at 10.1007/s12529-024-10343-w.

## Introduction

During the initial phase of the COVID-19 pandemic, countries worldwide relied on behavioral measures, such as social distancing, to control the spread of the virus [1]. This raises questions about how to balance the principle of beneficence, which encourages individuals to consider the needs and interests of others and take actions that promote the greater good and individual freedom, with individuals’ principled right to make choices and decisions that align with their values and interests, including the freedom to decide whether to contribute to a collective effort [2]. Preventive pandemic-related measures were considered a public good, while individuals who did not comply with these measures, sometimes called *free riders*, were considered an undeniable problem [3]. The free-rider effect can be seen as a conflict between individual freedom and the principle of beneficence. When individuals prioritize their freedom and act solely in their self-interest, they may act as free riders and thus benefit from collective efforts without contributing.

Research shows that the general population largely adhered to the stringent public health recommendations implemented during the COVID-19 pandemic [4]. However, studies have reported a detrimental impact on mental health due to these preventive measures [5]. Recent findings have emphasized the association between individual autonomy and better mental health outcomes [6]. Moreover, prosocial behavior has been linked to mental health, but only when individuals feel autonomous in their decision to engage in such behavior [7]. These findings raise concerns regarding the effectiveness of the behavioral measures that restrict contagion during the pandemic. Despite the comprehensive characteristics of pandemic restrictions, few studies have investigated the potential beneficial or non-beneficial effects of non-compliance, or “free-ridership.” Addressing this research gap is necessary for gaining additional insight into the consequences of non-compliance, which may, in turn, inform future cost-and-benefit analyses aimed at determining the common good in relation to future pandemics [8].

For two significant reasons, this study focuses on university students in Sweden, a young adult population of particular concern regarding pandemic-induced public health recommendations. Firstly, early adulthood is a transitional phase characterized by the prolonged exploration of identity and life goals [9], activities that were significantly disrupted by the pandemic. Secondly, because of this transition and exploration of life, university students can be considered a vulnerable group in that they exhibit a higher prevalence of mental disorders and an increased risk of experiencing suicidal thoughts [10]. With specific relevance to university students, this study focuses primarily on self-reported mental health and academic self-efficacy. Reviews of the initial phase of the pandemic, which involved lockdowns of university campuses and severe restrictions on social contacts in several countries, indicate an overall negative effect on students’ mental health, social life, and academic performance [11, 12]. Specifically, the negative effects identified in relation to mental health and academic self-efficacy were associated with a reduction in social interactions due to pandemic-induced public health restrictions [13].

The Swedish approach to managing the pandemic initially received considerable attention [14]. Based on a constitutional separation of government and technocracy, control measures were based on voluntary compliance and citizen responsibility [15]. To limit the spread of COVID-19, the Public Health Agency of Sweden [16] introduced a series of voluntary recommendations, including staying at home at the slightest symptom of infection, keeping a distance from others both indoors and outdoors, avoiding unnecessary visits to the elderly and people belonging to health risk groups, and avoiding public transportation and unnecessary travel. In Swedish university settings, cross-sectional studies conducted during the early phase of the pandemic confirmed negative effects on students’ mental health and academic self-efficacy [17, 18]. Most students complied with public health recommendations, where compliance was related to gender, age, and risk perception [18, 19].

The objective of the present study was to investigate the longitudinal effects of non-compliance with five public health recommendations on mental health and academic self-efficacy in Swedish university students. Specifically, the first aim was to estimate the effect of baseline non-compliance on mental health and academic self-efficacy 5 and 10 months post-baseline. The second aim was to estimate the effect of 5-month post-baseline non-compliance on mental health and academic self-efficacy at the 10-month follow-up.

## Methods

### Setting and Participants

This longitudinal study was conducted in Sweden during the enforced closure of university campuses, during a period when public health recommendations were applied. A baseline assessment was performed in May 2020, with follow-ups after 5 (October 2020) and 10 months (March 2021). The Swedish Public Health Agency [16] has reported that COVID-19 was introduced in early February 2020, with a steady increase in infection rates during the spring, followed by further intense waves during the winter and spring of 2021. Thus, these three time points correspond roughly to the pandemic’s three first major waves in Sweden.

At baseline, students responded to announcements on the websites of ten public universities, colleges, and the National Association of Student Unions. Interested students followed a URL or a QR code to obtain detailed information. After having provided digital informed consent, a short online survey was administered. A total of 4495 consents were recorded from students at 19 universities and university colleges, of which 3123 (69.5%) consented to follow-ups distributed by email. Responses to the 5-month follow-up were received from 1818 (58.2%) participants, and responses to the 10-month follow-up were obtained from 1657 (53.1%) participants. The study was pre-approved by the Swedish Ethical Review Authority (Ref. No. 2020–02109), and participants received no compensation.

### Measures

Participants were asked to rate their compliance (often, very often or always [coded as yes] or less often, seldom or never [coded as no]) during the past 4 weeks on the following five public health recommendations stipulated by the Swedish Public Health Agency: (1) “Remained at home,” (2) “Kept a distance from others when you have gone out,” (3) “Avoided meeting with persons who are older/in a risk group,” (4) “Avoided traveling with public transportation,” and (5) “Avoided travel to other places in Sweden.” Mental health was assessed with the following question “How has your mental health been affected by the COVID-19 pandemic during the past 4 weeks?” where the four response alternatives were as follows: “No effect,” “My mental health has been worse,” “My mental health has been better,” and “My mental health has been both better and worse.” Finally, academic self-efficacy was assessed with the question “How have your studies been going during the past 4 weeks?” where the five response alternatives were as follows: “No change, my studies are going as usual,” “My studies have been going worse,” “My studies have been going better,” “My studies have been going both better and worse,” and “I am not studying at this time.”

### Analysis

Pearson’s chi-square test was used to compare the proportion of non-compliers at baseline and the 5-month follow-up and ratings of mental health and academic self-efficacy at the 5- and 10-month follow-ups.

In this study, 30 estimands of interest related to students’ non-compliance with public health recommendations during the COVID-19 pandemic were available. The effects of baseline non-compliance with the five public health recommendations on mental health and academic self-efficacy were measured at the 5- and 10-month follow-ups. The effects of non-compliance on mental health and academic self-efficacy at the 5-month follow-up with each of the five public health recommendations were measured at the 10-month follow-up. A directed acyclic graph (Online supplementary Fig. 1) depicts causal assumptions formulated by the authors to estimate effects from observational data. The assumed causal links were used with Pearl’s do-calculus [20] to identify necessary adjustment variables. When analyzing each effect estimand related to baseline compliance factors, models were adjusted for age, gender, and non-compliance with the public health recommendations not considered as focal exposure, i.e., handwashing and sneezing/coughing in one’s sleeve. Similarly, when evaluating the effect estimands involving 5-month compliance factors, models were adjusted for age, gender, mental health changes, academic self-efficacy reported at baseline, and the public health recommendations.

Multilevel multinomial regression was used to estimate effects, with adaptive intercepts for universities and subjects in longitudinal models. Models were estimated using Bayesian inference [21]. Covariates and adaptive intercepts were given standard normal priors, and posterior distributions were used as point estimates of effects alongside 2.5% and 97.5% posterior percentiles, representing a 95% comparability interval (CI). The posterior probability of effect estimates less than or greater than the null is also reported.

## Results

Online Supplementary Table 1 shows the distributions of self-reports regarding non-compliance with COVID-19 public health recommendations at baseline and 5 months after the baseline assessment, cross-tabulated with self-reported change in mental health at the 5- and 10-month follow-ups. For each public health recommendation, the right side of the table displays chi-square statistics. It compares the proportion of baseline non-compliance at the 5-month assessment (A) with that of baseline non-compliance at the 10-month assessment (B). Subsequently, the proportion of baseline non-compliance in these two groups is compared with 5-month non-compliance at the 10-month assessment (C). In the context of mental health and relative to baseline compliance, the ratings at the 5-month assessment (a) are compared with those at the 10-month assessment (b). Following this, the ratings in these two groups are compared with the 10-month ratings relative to 5-month compliance. Online Supplementary Table 2 presents the corresponding cross-tabulations and chi-square statistics for changes in academic self-efficacy.

Concerning all the public health recommendations studied and across both follow-ups, a minority of students were non-compliant compared to those who were compliant. When comparing the proportions of non-compliers at the baseline assessment (A and B) and those at the 10-month assessment (C), the test statistics show that non-compliance increased significantly over time. The test statistics also show that mental health and academic self-efficacy ratings changed between the 5-month and 10-month follow-up. Regarding mental health at the 5-month follow-up (Online Supplementary Table 1), the most frequent answer was “no change” and “worse,” followed by “both better and worse,” and “better.” At the 10-month follow-up, “worse” was the most frequent answer, followed by “both better and worse,” “no change,” and “better.” Concerning academic self-efficacy at the 5-month follow-up (Online Supplementary Table 2), the most frequent answer was “both better and worse,” followed by “worse,” “no change,” “not studying,” and “better.” At the 10-month follow-up, “worse” was the most frequent answer, followed by “both better and worse,” “no change,” “not studying,” and “better.”

### Did Non-compliance Influence Mental Health?

**Fig. 1:**
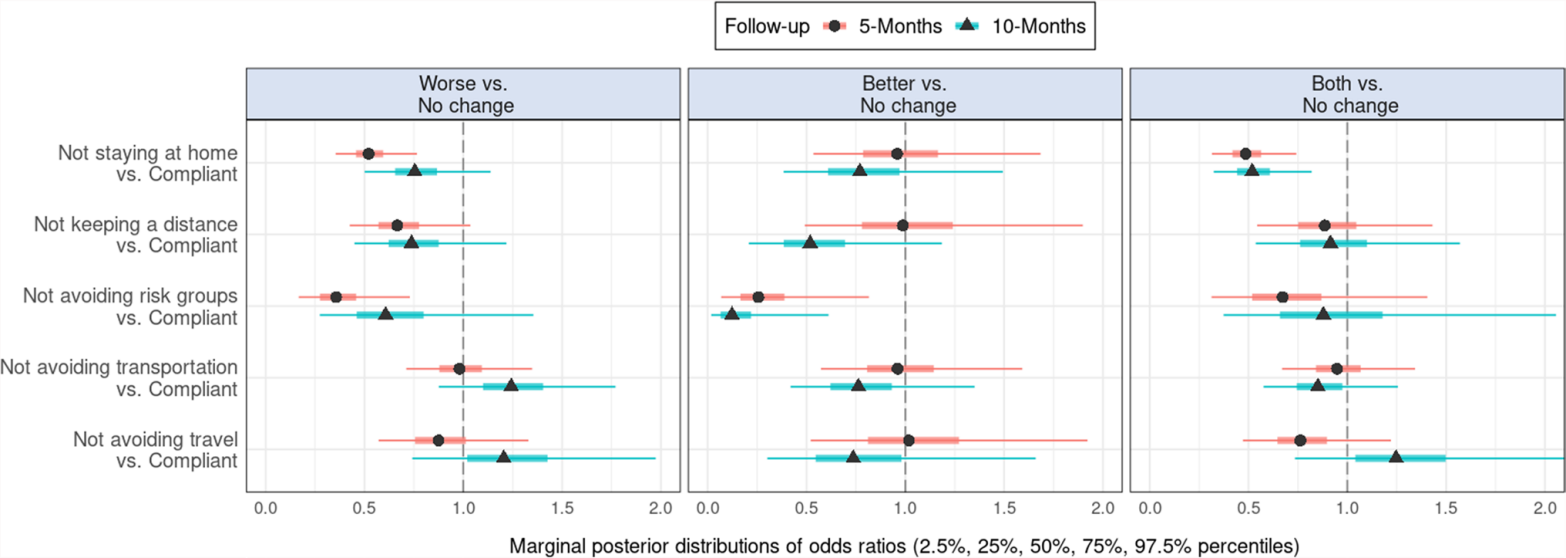
Marginal posterior distributions of coefficients in the multinomial regression models estimating effects of non-compliance with COVID-19 public health recommendations at baseline on self-reported change in mental health at 5 months and 10 months follow-ups

**Fig. 2:**
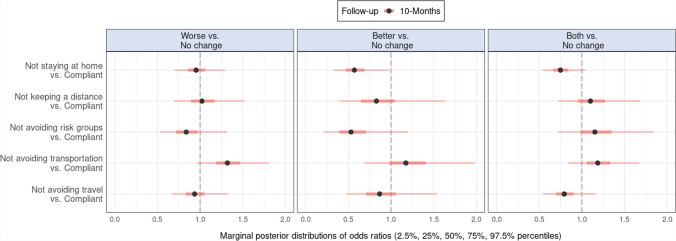
Marginal posterior distributions of coefficients in the multinomial regression models estimating effects of non-compliance with COVID-19 public health recommendations at 5 months follow-up on self-reported change in mental health at 10 months follow-up

Figure 1 presents marginal posterior distributions of coefficients in the multilevel regression models estimating the effects of students’ non-compliance with five public health recommendations on self-reported change in mental health at 5-month and 10-month follow-ups. Figure 2 presents the corresponding effect estimates of non-compliance at 5-month follow-up on change in mental health at 10-month follow-up. Online Supplementary Table 3 provides numerical details.

For the public health recommendation of “staying at home,” non-compliance at baseline was associated with lower odds of reporting worse mental health rather than no change at the 5-month follow-up. Baseline non-compliance with “staying at home” was also associated with lower odds of reporting both better and worse mental health rather than no change at the 5- and 10-month follow-ups (see Fig. 1). In contrast, non-compliance with “staying at home” at the 5-month follow-up was associated with lower odds of reporting better mental health rather than no change at the 10-month follow-up (see Fig. 2).

Like “staying at home,” non-compliance at baseline with the public health recommendation to “avoid risk groups” was associated with lower odds of reporting worse mental health rather than no change at the 5-month follow-up (Fig. 1). In contrast, baseline non-compliance with avoiding risk groups was associated with lower odds of reporting better mental health rather than no change at the 5- and 10-month follow-ups (Fig. 1). The estimates showed considerable uncertainty for the remaining associations between recommendations and outcomes.

### Did Non-compliance Influence Academic Self-efficacy?

**Fig. 3:**
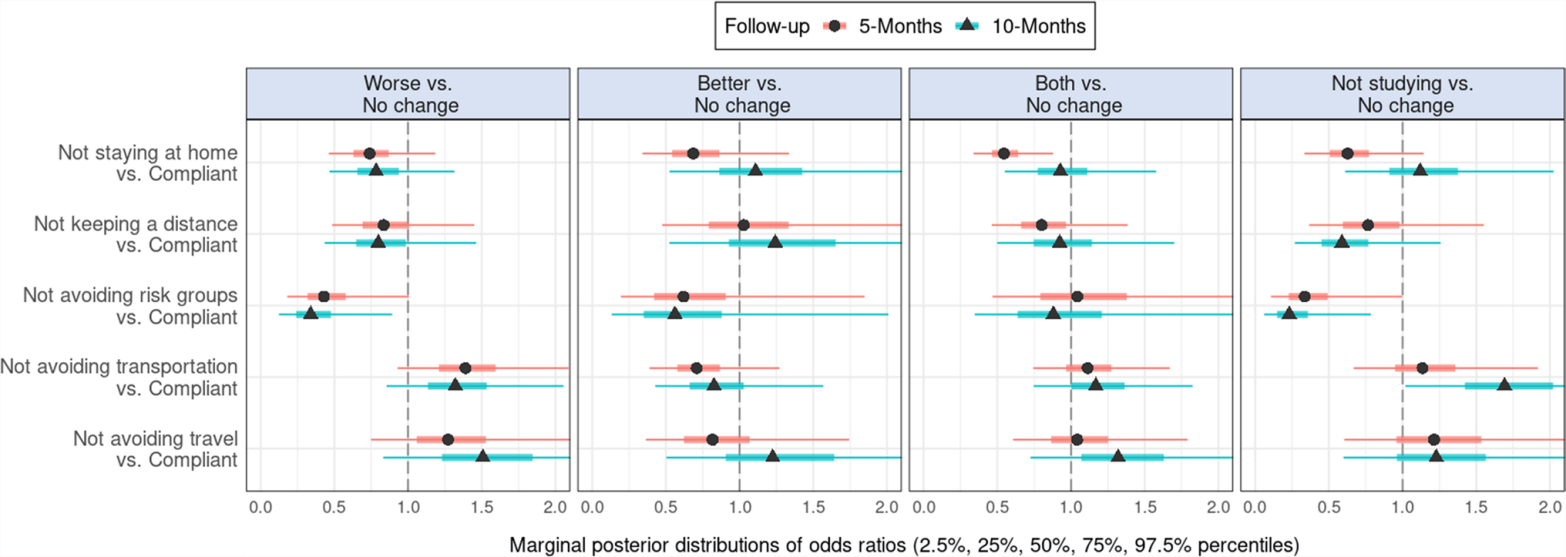
Marginal posterior distributions of coefficients in the multinomial regression models estimating effects of non-compliance with COVID-19 public health recommendations at baseline on self-reported change in academic self-efficacy at 5 months and 10 months follow-ups

**Fig. 4:**
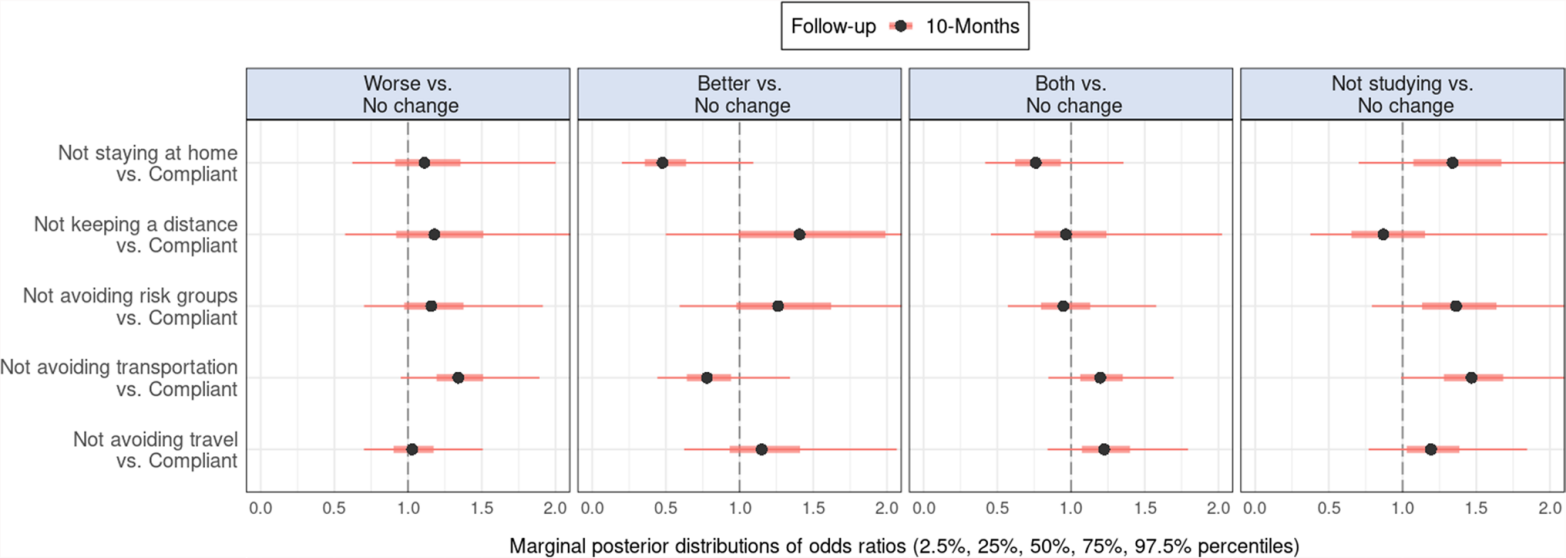
Marginal posterior distributions of coefficients in the multinomial regression models estimating effects of non-compliance with COVID-19 public health recommendations at 5 months follow-up on self-reported change in academic self-efficacy at 10 months follow-up

The same analytic procedures were used to assess whether baseline non-compliance affected academic self-efficacy at 5- and 10-month follow-ups (Fig. 3) and whether 5-month non-compliance affected academic self-efficacy at the 10-month follow-up (Fig. 4). Online Supplementary Table 4 shows the numerical details.

Non-compliance with the recommendation of “staying at home” at baseline was associated with lower odds of reporting both better and worse academic self-efficacy, rather than no change, at the 5-month follow-up. Non-compliance with the recommendation to “avoid risk groups” at baseline was also associated with lower odds of reporting worse academic self-efficacy rather than no change at the 10-month follow-up and lower odds of reporting not studying rather than no change at the 10-month follow-up. Finally, non-compliance at baseline with the recommendation of “avoiding transportation” was associated with increased odds of reporting not studying rather than no change in academic self-efficacy at the 10-month follow-up (Fig. 3). The estimates showed considerable uncertainty for the remaining associations between recommendations and outcomes.

## Discussion

With all public health recommendations studied and across follow-ups, a minority of students were non-compliant compared to those who complied. The proportion of non-compliers increased between the baseline assessment and the 5-month follow-up. These findings are consistent with previous studies [4, 18, 19, 22]. Moreover, the present longitudinal study showed few differences between compliant and non-compliant students in terms of effects on mental health and academic self-efficacy. Most of the strongly associated findings indicated that the group commonly referred to as “free riders” experienced fewer negative effects. However, most associations were not strong, and the estimated uncertainty was high.

Findings related to the “non-studying” category suggest that free riders were less likely to be concerned about risk groups. Additionally, avoiding public transportation involved a more significant challenge for those who can be assumed to have been working instead of continuing to study at the university. One possible explanation is that universities were under lockdown during the pandemic; this did not apply to all workplaces, although workers who could work from home were encouraged to do so. It remains unclear whether the “non-studying” category includes students who left higher education due to the pandemic because they had completed their studies or had other reasons for not studying. However, previous reports have indicated that university dropout rates during the pandemic were associated with self-contagion [18].

While there were no discernible longitudinal effects explicitly linked to non-compliance with maintaining a distance from others and avoiding unnecessary travel, it is worth noting that a comparable number of students demonstrated non-adherence to these recommendations—as for the remaining public health recommendations. Prior research has shown that barriers to adhering to public health guidance often revolve around self-experienced needs, such as seeking or providing support from family and friends to counteract social isolation or to address mental health concerns [23]. Although it was relatively easy to maintain physical distance during the pandemic, these findings suggest that non-compliance dynamics may be more complex.

The increase in non-compliance over time, i.e., between the baseline and 5-month assessments, can be explained by students becoming increasingly tired of following public health recommendations. This phenomenon is often referred to as “pandemic fatigue” or “compliance fatigue” [22]. This psychological response can occur when individuals feel worn out, demotivated, or less willing to continue following recommended health measures over an extended period.

Different psychological theories and models have been used to explain why individuals did or did not comply with public health recommendations during the COVID-19 pandemic. These include the social norms theory [24], the self-determination theory [25], the cognitive dissonance theory [26], the conservation of resources theory [27], and the health beliefs model [28].

Although not explicitly tested, the present results can be interpreted using Self-Determination Theory (SDT) [29] as a framework. While SDT does not explicitly address issues of beneficence and individual freedom, it does include related concepts. According to SDT, individuals who feel connected to others are likelier to engage in behaviors that benefit others and contribute to the greater good. Similarly, individuals with a sense of autonomy and the freedom to make choices are more likely to be motivated and experience personal agency. SDT assumes that motivation plays a significant role in human behavior. Specifically, SDT distinguishes between intrinsic and extrinsic motivation, i.e., differences between experiencing oneself as motivated due to the satisfaction of engaging in an activity (intrinsic motivation) versus being motivated by external factors, including, e.g., rewards or punishment (extrinsic motivation). The “free-rider effect” identified in the present study may result from an interplay between intrinsic and extrinsic motivation. Specifically, non-compliant individuals early in the pandemic may have been motivated by intrinsic personal values and interests, resulting in positive outcomes. In contrast, non-compliant individuals later in the pandemic were perhaps motivated by external factors, such as meeting expectations and avoiding negative consequences during the initial phases of the pandemic, and later by compliance fatigue.

Following national laws and regulations, the Swedish approach was based on voluntary public health recommendations, which allowed individuals to make informed personal decisions. The present findings show that students effectively balanced the principles of beneficence and autonomy. The voluntary approach resulted in marginal non-compliance during the initial phase of the pandemic, where the free riders experienced some beneficial effects on mental health and academic self-efficacy. This finding underscores the multifaceted characteristics of individual responses to preventive measures and highlights the importance of considering diverse factors that influence the outcomes. Rather than adopting a one-size-fits-all approach, tailored interventions appear necessary to enhance public health compliance effectively. Moreover, the results point to the potential for individuals to make informed decisions that mitigate adverse impacts on their mental health.

In addition, while the Swedish approach involved maintaining personal freedom, research shows that its voluntary aspects were associated with lower excess mortality compared to many other European countries [30]. Since a more aggressive approach with a wide range of stringent interventions, some of which entail limiting civil rights, has been associated with more confirmed cases, higher fatality rates, and substantial social and economic costs, it has been suggested that stringent restrictive policies may be less helpful [31].

For future pandemics comparable to COVID-19, the findings suggest that a voluntary approach that provides opportunities to balance the principles of beneficence and autonomy seems optimal for two reasons. First, university students in Sweden are responsive to public health recommendations; second, the possibility of making autonomous decisions may involve some flexibility for the minority of students needing a sense of autonomy to cope with their mental health and academic self-efficacy.

### Strengths and Limitations

This study was initiated as a prompt response to investigate any systematic changes during the pandemic. To reduce students’ response burden during remote education [32], the online questionnaire was brief and focused on its design. Consequently, the control variables available for the current analysis may have limitations since they do not include all variables potentially associated with adherence to public health recommendations [33], mental health [34], and academic self-efficacy [35].

The primary strength of the current study is its longitudinal design, coupled with the methodological approach of studying causal effects based on the assumptions shown in a predefined directive acyclic graph (DAG) and the use of do-calculus to identify and adjust for confounding factors [20]. The causal graph implies that findings can be limited by the graph’s representativeness of casual relationships. If the predefined relationships do not hold, the effect estimates may still be confounded and should be interpreted as associations. The analytic model does not protect against spurious associations induced by collider bias, apart from where time can guarantee the direction of causality. Due to this, the estimate associations presented should only be understood to represent effects if the causal model is appropriate. In addition, there was substantial uncertainty in many of the estimated associations, which means that findings should be considered given the uncertainty with which we can conclude the data.

It should be noted that the current study includes a limited group of students from a limited number of universities who chose to participate after responding to an advertisement. This means the participants may not fully represent the total group of students, a possibility discussed in a previous publication from our research group [18]. This suggests that the risk of a significant lack of representativity was lower rather than higher. It should also be noted that the findings may be affected by non-response concerning the two follow-ups.

Among possible sources of bias (including self-report bias, social desirability bias, recall bias, and measurement error bias) that may have affected the present study, the 41.8–46.9% attrition rates may be the most prominent problem as they may contribute to reduced generalizability [36]. As identified in a study investigating attrition from longitudinal studies conducted during the COVID-19 pandemic [37], the attrition rates in this study may be related to the variables of interest. This may, in turn, result in over- or underestimations.

Aligning with the need to launch the study quickly, the measures used were not validated a priori. However, they were conceived with the help of a student in clinical psychology, who was a co-author for the first study published from these data and whose contribution has after that been acknowledged. The items were thus co-created from joint student-researcher perspectives. The study evaluated the five public health recommendations the Public Health Agency of Sweden communicated throughout the pandemic. Despite extensive public health awareness campaigns, individuals may still have interpreted the public health recommendations and the questions relating to these in different ways. This may, in turn, have influenced self-reports of compliance.

Single-item measures were used to assess the two constructs of interest, as single items are considered superior for reducing the response burden [32]. While it should be acknowledged that single items may have lower content validity, sensitivity, and reliability compared to multiple-item measures, single-item measures can still effectively replace multiple-item measures. Importantly, research has demonstrated that single-item measures are valid and reliable for mental health assessment [38, 39] and self-efficacy [40, 41]. However, it should be recognized that the single-item measure of self-efficacy was designed to assess the specific and changeable characteristics of the construct [42], thus departing from the conventional approach of treating self-efficacy as a manifest and global state [43]. Research shows that validated multiple-item instruments strongly correlate with academic self-efficacy and mental health [44]. Despite lacking a priori item validation, the present study shows similar associations between academic self-efficacy and mental health, supporting criterion validity [18]. On the other hand, since construct validity is lacking, an alternative is to consider what is being addressed as “mental health” as “experienced changes in mental health” and “academic self-efficacy” as “experiences of how studies were going.” We perceive that face validity was high given the number and proportion of student respondents at baseline and follow-up time points. However, we acknowledge that content validity is limited with single-item measures, given the potential inability of single items to cover the entire domain of interest fully. Thus, future research would be needed to establish the proper reliability and validity of the single-item measures used in this study [32].

## Conclusion

In conclusion, this longitudinal study investigated the effects of non-compliance with public health recommendations on mental health and academic self-efficacy among university students during the COVID-19 pandemic in Sweden. Overall, the number of students who complied outweighed the minority who were non-compliant, but the proportion of non-compliers increased over time. The findings suggest some evidence that non-compliants experienced fewer mental health effects and worsening academic self-efficacy. Nonetheless, these findings should be interpreted while considering the study’s limitations.

Addressing the research gap on the effects of non-compliance with contagion-related recommendations during public health crises is essential to inform decision-making and promote the common good. These insights emphasize the complex dynamics between individual autonomy and collectivistic values and may guide future strategies to manage pandemics effectively.

## Supplementary Information

Below is the link to the electronic supplementary material.

Supplementary file1 Online Supplementary Figure 1 Directed Acyclic Graph (DAG) showing causal assumptions to estimate the effects (PNG 209 KB)

Supplementary file2 (DOCX 25 KB)

Supplementary file3 (DOCX 26 KB)

Supplementary file4 (DOCX 17 KB)

Supplementary file5 (DOCX 17 KB)

## Data Availability

The dataset analyzed in the current study is available from the corresponding author upon reasonable request.
