# Supplementary figures and images for: Non-compliance with COVID-19 Health Recommendations: Five- and Ten-Month Effects on Mental Health and Academic Self-efficacy Among University Students in Sweden
Source: Int J Behav Med. 2024 Dec 30;33(3):452–60. doi: 10.1007/s12529-024-10343-w (PMC13342285; doi:10.1007/s12529-024-10343-w)

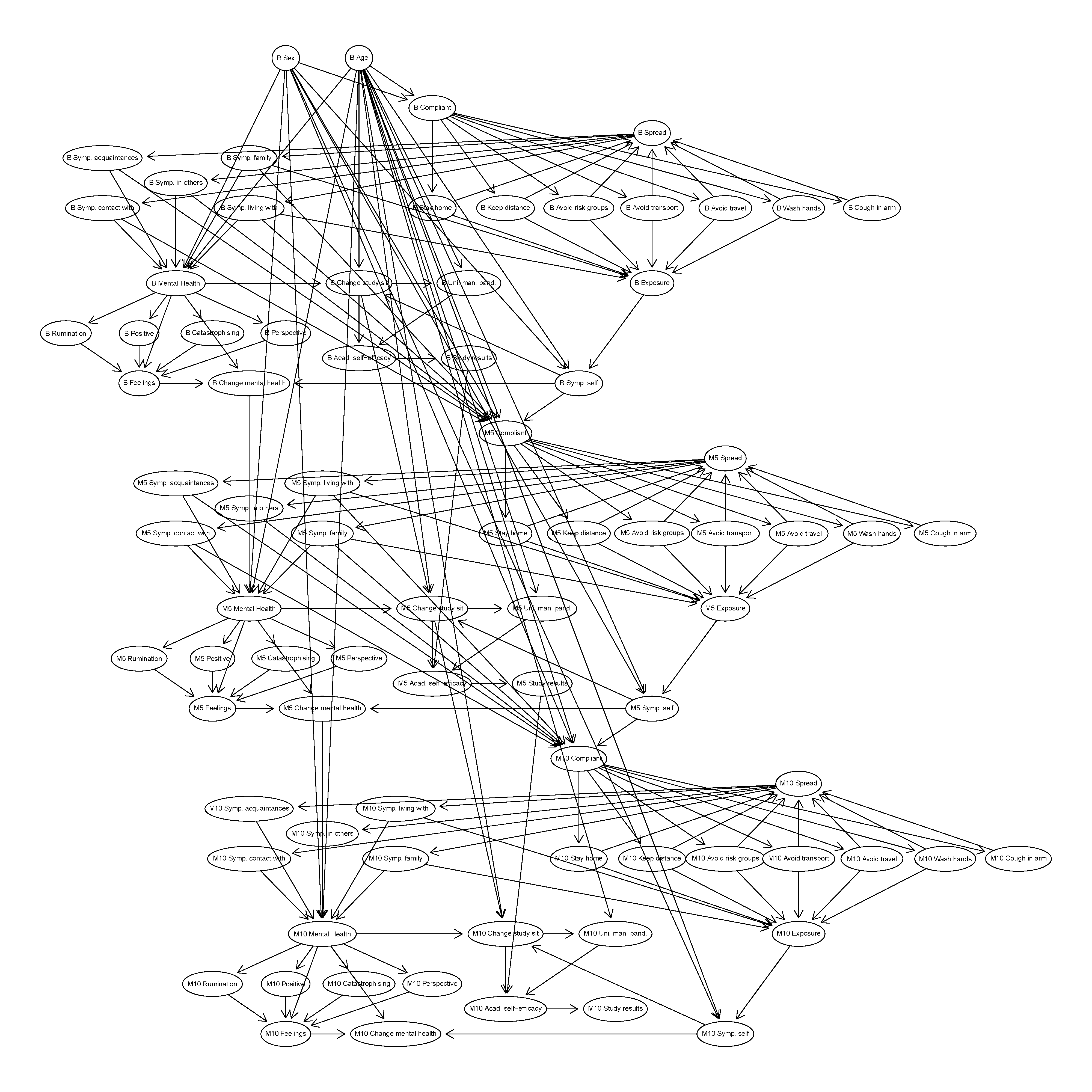

Supplement: Supplementary file 1 — Supplementary file1 Online Supplementary Figure 1 Directed Acyclic Graph (DAG) showing causal assumptions to estimate the effects (PNG 209 KB) [file 12529_2024_10343_MOESM1_ESM.png]
