# Supplementary material for: Non-compliance with COVID-19 Health Recommendations: Five- and Ten-Month Effects on Mental Health and Academic Self-efficacy Among University Students in Sweden
Source: Int J Behav Med. 2024 Dec 30;33(3):452–60. doi: 10.1007/s12529-024-10343-w (PMC13342285; doi:10.1007/s12529-024-10343-w)
Supplement: Supplementary file 2 — Supplementary file2 (DOCX 25 KB) [file 12529_2024_10343_MOESM2_ESM.docx]

**Online Supplementary Table 1.** Contingency table (frequency) showing the distribution of self-reports regarding compliance with COVID-19 public health recommendations, at baseline and five months after the baseline assessment, in relation to self-reported change in mental health at the 5- and 10-month follow-ups.

|  | | | Self-reported change in mental health | | | | | | | | | | | Chi2 statistics | | | |
| --- | --- | --- | --- | --- | --- | --- | --- | --- | --- | --- | --- | --- | --- | --- | --- | --- | --- |
|  |  |  | 5-month follow-up | | | | | 10-month follow-up | | | | | | Compliance (df=2) | | Mental health (df=3) | |
|  |  |  | No change | Worse | Better | Both | Total (%) ^A^ |  | No change | Worse | Better | Both | Total (%) ^B^ | A vs B |  | a vs b |  |
| Compliance with Covid-19 public health recommendations at baseline | Staying at home | Yes | 504 | 552 | 84 | 361 | 1501 (82.6) | Yes | 299 | 606 | 82 | 382 | 1369 (82.6) | 0.00 |  | 69.82* |  |
|  |  | No | 144 | 93 | 23 | 56 | 316 (17.4) | No | 81 | 130 | 16 | 61 | 288 (17.4) |  |  |  |  |
|  |  | Total (%) ^a^ | 648 (35.6) | 645 (35.5) | 107 (5.9) | 417 (23.0) | 1817 (100) | Total (%) ^b^ | 380 (22.9) | 736 (44.4) | 98 (5.9) | 443 (26.8) | 1657 (100) |  |  |  |  |
|  | Keeping a distance | Yes | 567 | 580 | 93 | 373 | 1613 (88.7) | Yes | 333 | 659 | 91 | 395 | 1478 (89.2) | 0.20 |  | 69.82* |  |
|  |  | No | 81 | 65 | 14 | 45 | 205 (11.3) | No | 47 | 77 | 7 | 48 | 179 (10.8) |  |  |  |  |
|  |  | Total (%) ^a^ | 648 (35.6) | 645 (35.5) | 107 (5.9) | 418 (23.0) | 1818 (100) | Total (%) ^b^ | 380 (22.9) | 736 (44.4) | 98 (5.9) | 443 (26.8) | 1657 (100) |  |  |  |  |
|  | Avoiding risk groups | Yes | 610 | 633 | 106 | 405 | 1754 (96.5) | Yes | 362 | 715 | 98 | 427 | 1602 (96.7) | 0.11 |  | 69.82* |  |
|  |  | No | 38 | 12 | 1 | 13 | 64 (3.5) | No | 18 | 21 | 0 | 16 | 55 (3.3) |  |  |  |  |
|  |  | Total (%) ^a^ | 648 (35.6) | 645 (35.5) | 107 (5.9) | 418 (23.0) | 1818 (100) | Total (%) ^b^ | 380 (22.9) | 736 (44.4) | 98 (5.9) | 443 (26.8) | 1657 (100) |  |  |  |  |
|  | Avoiding transportation | Yes | 443 | 449 | 72 | 293 | 1257 (69.2) | Yes | 264 | 491 | 73 | 320 | 1148 (69.3) | 0.00 |  | 69.31* |  |
|  |  | No | 204 | 195 | 35 | 125 | 559 (30.8) | No | 116 | 244 | 25 | 123 | 508 (30.7) |  |  |  |  |
|  |  | Total (%) ^a^ | 647 (35.6) | 644 (35.5) | 107 (5.9) | 418 (23.0) | 1816 (100) | Total (%) ^b^ | 380 (22.9) | 735 (44.4) | 98 (5.9) | 443 (26.8) | 1656 (100) |  |  |  |  |
|  | Avoiding travel | Yes | 560 | 562 | 92 | 371 | 1585 (97.4) | Yes | 339 | 636 | 90 | 385 | 1450 (87.7) | 0.07 |  | 69.25* |  |
|  |  | No | 87 | 80 | 15 | 46 | 228 (12.6) | No | 41 | 96 | 8 | 58 | 203 (12.3) |  |  |  |  |
|  |  | Total (%) ^a^ | 647 (35.7) | 642 (35.4) | 107 (5.9) | 417 (23.0) | 1813 (100) | Total (%) ^b^ | 380 (23.0) | 732 (44.3) | 98 (5.9) | 443 (26.8) | 1653 (100) |  |  |  |  |
| Compliance with Covid-19 public health recommendations at 5 months post-baseline |  |  |  |  |  |  |  |  | No change | Worse | Better | Both | Total (%) ^C^ | A vs C | B vs C | a vs c | b vs c |
|  | Staying at home | Yes |  |  |  |  |  |  | 187 | 368 | 59 | 248 | 862 (59.6) | 213.18* | 264.45* | 62.42* | 0.04 |
|  |  | No |  |  |  |  |  |  | 148 | 275 | 25 | 136 | 584 (40.4) |  |  |  |  |
|  |  | Total (%) ^c^ |  |  |  |  |  |  | 335 (23.2) | 643 (44.5) | 84 (5.8) | 384 (26.5) | 1446 (100) |  |  |  |  |
|  | Keeping a distance | Yes |  |  |  |  |  |  | 284 | 529 | 74 | 314 | 1201 (82.9) | 23.02* | 25.98* | 62.75* | 0.03 |
|  |  | No |  |  |  |  |  |  | 51 | 116 | 11 | 70 | 248 (17.1) |  |  |  |  |
|  |  | Total (%) ^c^ |  |  |  |  |  |  | 335 (23.1) | 645 (44.5) | 85 (5.9) | 384 (26.5) | 1449 (100) |  |  |  |  |
|  | Avoiding risk groups | Yes |  |  |  |  |  |  | 285 | 578 | 79 | 334 | 1276 (88.3) | 81.15* | 80.83* | 64.06* | 0.01 |
|  |  | No |  |  |  |  |  |  | 47 | 66 | 6 | 50 | 169 (11.7) |  |  |  |  |
|  |  | Total (%) ^c^ |  |  |  |  |  |  | 332 (23.0) | 644 (44.6) | 85 (5.9) | 384 (26.6) | 1445 (100) |  |  |  |  |
|  | Avoiding transportation | Yes |  |  |  |  |  |  | 208 | 330 | 50 | 215 | 803 (55.4) | 65.92* | 63.99* | 62.63* | 0.03 |
|  |  | No |  |  |  |  |  |  | 127 | 315 | 35 | 169 | 646 (44.6) |  |  |  |  |
|  |  | Total (%) ^c^ |  |  |  |  |  |  | 335 (23.1) | 645 (44.5) | 85 (5.9) | 384 (26.5) | 1449 (100) |  |  |  |  |
|  | Avoiding travel | Yes |  |  |  |  |  |  | 252 | 483 | 64 | 301 | 1100 (76.2) | 70.23* | 70.60* | 62.34* | 0.06 |
|  |  | No |  |  |  |  |  |  | 83 | 159 | 20 | 82 | 344 (23.8) |  |  |  |  |
|  |  | Total (%) ^c^ |  |  |  |  |  |  | 335 (23.2) | 642 (44.5) | 84 (5.8) | 383 (26.5) | 1444 (100) |  |  |  |  |

Notes.

Compliance at baseline in students responding to the 5-month follow-up (A) and to the 10-month follow-up (B).

Compliance at 5 months post-baseline in students responding to the 10-month follow-up (C).

Self-reported change in mental health at the 5-month follow-up (a & b) and at the 10-month follow-up (c).

df = degrees of freedom.

*p < .05
