# Supplementary material for: Non-compliance with COVID-19 Health Recommendations: Five- and Ten-Month Effects on Mental Health and Academic Self-efficacy Among University Students in Sweden
Source: Int J Behav Med. 2024 Dec 30;33(3):452–60. doi: 10.1007/s12529-024-10343-w (PMC13342285; doi:10.1007/s12529-024-10343-w)
Supplement: Supplementary file 3 — Supplementary file3 (DOCX 26 KB) [file 12529_2024_10343_MOESM3_ESM.docx]

**Online Supplementary Table 2.** Contingency table (frequency) showing the distribution of self-reports regarding compliance with COVID-19 public health recommendations, at baseline and five months after the baseline assessment, in relation to self-reported change in academic self-efficacy at the 5- and 10-month follow-ups.

|  | | | Self-reported change in academic self-efficacy | | | | | | | | | | | | | Chi2 statistics | | | |
| --- | --- | --- | --- | --- | --- | --- | --- | --- | --- | --- | --- | --- | --- | --- | --- | --- | --- | --- | --- |
|  |  |  | 5-month follow-up | | | | | | 10-month follow-up | | | | | | | Compliance (df=2) | | Self-efficacy (df=4) | |
|  |  |  | No change | Worse | Better | Both | Not  studying | Total (%) ^A^ |  | No change | Worse | Better | Both | Not  studying | Total (%) ^B^ | A vs B |  | a vs b |  |
| Compliance with Covid-19 public health recommendations at baseline | Staying at home | Yes | 279 | 467 | 100 | 491 | 147 | 1484 (82.6) | Yes | 269 | 427 | 89 | 374 | 197 | 1356 (82.5) | 0.01 |  | 25.01* |  |
|  |  | No | 80 | 103 | 18 | 86 | 26 | 313 (17.4) | No | 54 | 86 | 20 | 81 | 47 | 288 (17.5) |  |  |  |  |
|  |  | Total (%) ^a^ | 359 (20.0) | 570 (31.7) | 118 (6.6) | 577 (32.1) | 173 (9.6) | 1797 (100) | Total (%) ^b^ | 323 (19.6) | 513 (31.2) | 109 (6.6) | 455 (27.7) | 244 (14.9) | 1644 (100) |  |  |  |  |
|  | Keeping a distance | Yes | 314 | 505 | 104 | 515 | 156 | 1594 (88.7) | Yes | 288 | 453 | 95 | 405 | 225 | 1466 (89.2) | 0.23 |  | 24.73* |  |
|  |  | No | 45 | 65 | 14 | 63 | 17 | 204 (11.3) | No | 36 | 60 | 14 | 50 | 18 | 178 (10.8) |  |  |  |  |
|  |  | Total (%) ^a^ | 359 (20.0) | 570 (31.7) | 118 (6.6) | 578 (32.1) | 173 (9.6) | 1798 (100) | Total (%) ^b^ | 324 (19.7) | 513 (31.2) | 109 (6.6) | 455 (27.7) | 243 (14.8) | 1644 (100) |  |  |  |  |
|  | Avoiding risk groups | Yes | 339 | 556 | 115 | 554 | 170 | 1734 (96.4) | Yes | 305 | 501 | 106 | 437 | 240 | 1589 (96.7) | 0.12 |  | 25.15* |  |
|  |  | No | 20 | 14 | 3 | 24 | 3 | 64 (3.6) | No | 18 | 12 | 3 | 18 | 4 | 55 (3.3) |  |  |  |  |
|  |  | Total (%) ^a^ | 359 (20.0) | 570 (31.7) | 118 (6.6) | 578 (32.1) | 173 (9.6) | 1798 (100) | Total (%) ^b^ | 323 (19.6) | 513 (31.2) | 109 (6.6) | 455 (27.7) | 244 (14.9) | 1644 (100) |  |  |  |  |
|  | Avoiding transportation | Yes | 255 | 373 | 89 | 402 | 122 | 1241 (69.1) | Yes | 238 | 344 | 82 | 316 | 160 | 1140 (69.4) | 0.02 |  | 24.65* |  |
|  |  | No | 105 | 196 | 29 | 175 | 50 | 555 (30.9) | No | 86 | 169 | 27 | 139 | 82 | 503 (30.6) |  |  |  |  |
|  |  | Total (%) ^a^ | 360 (20.0) | 569 (31.7) | 118 (6.6) | 577 (32.1) | 172 (9.6) | 1796 (100) | Total (%) ^b^ | 324 (19.7) | 513 (31.2) | 109 (6.6) | 455 (27.7) | 242 (14.8) | 1643 (100) |  |  |  |  |
|  | Avoiding travel | Yes | 317 | 488 | 107 | 505 | 150 | 1567 (87.4) | Yes | 291 | 437 | 96 | 398 | 216 | 1438 (87.7) | 0.06 |  | 24.53* |  |
|  |  | No | 42 | 80 | 11 | 71 | 22 | 226 (12.6) | No | 32 | 74 | 13 | 57 | 26 | 202 (12.3) |  |  |  |  |
|  |  | Total (%) ^a^ | 359 (20.0) | 568 (31.7) | 118 (6.6) | 576 (32.1) | 172 (9.6) | 1793 (100) | Total (%) ^b^ | 323 (19.7) | 511 (31.2) | 109 (6.6) | 455 (27.7) | 242 (14.8) | 1640 (100) |  |  |  |  |
| Compliance with Covid-19 public health recommendations at 5 months post-baseline |  |  |  |  |  |  |  |  |  | No change | Worse | Better | Both | Not  studying | Total (%) ^C^ | A vs C | B vs C | a vs c | b vs c |
|  | Staying at home | Yes |  |  |  |  |  |  |  | 169 | 264 | 62 | 239 | 120 | 854 (59.5) | 212.96* | 334.94* | 27.98* | 0.47 |
|  |  | No |  |  |  |  |  |  |  | 113 | 195 | 30 | 146 | 98 | 582 (40.5) |  |  |  |  |
|  |  | Total (%) ^c^ |  |  |  |  |  |  |  | 282 (19.6) | 459 (32.0) | 92 (6.4) | 385 (26.8) | 218 (15.2) | 1436 (100) |  |  |  |  |
|  | Keeping a distance | Yes |  |  |  |  |  |  |  | 239 | 370 | 77 | 322 | 186 | 1194 (83.0) | 21.58* | 24.90* | 28.18* | 0.46 |
|  |  | No |  |  |  |  |  |  |  | 43 | 89 | 15 | 65 | 33 | 245 (17.0) |  |  |  |  |
|  |  | Total (%) ^c^ |  |  |  |  |  |  |  | 282 (19.6) | 459 (31.9) | 92 (6.4) | 387 (26.9) | 219 (15.2) | 1439 (100) |  |  |  |  |
|  | Avoiding risk groups | Yes |  |  |  |  |  |  |  | 249 | 408 | 80 | 349 | 182 | 1268 (88.4) | 78.50* | 78.75* | 27.26* | 0.34 |
|  |  | No |  |  |  |  |  |  |  | 33 | 49 | 12 | 38 | 35 | 167 (11.6) |  |  |  |  |
|  |  | Total (%) ^c^ |  |  |  |  |  |  |  | 282 (19.7) | 457 (31.8) | 92 (6.4) | 387 (27.0) | 217 (15.1) | 1435 (100) |  |  |  |  |
|  | Avoiding transportation | Yes |  |  |  |  |  |  |  | 182 | 227 | 63 | 211 | 117 | 800 (55.6) | 62.56* | 62.55* | 28.03* | 0.45 |
|  |  | No |  |  |  |  |  |  |  | 101 | 232 | 29 | 176 | 101 | 639 (44.4) |  |  |  |  |
|  |  | Total (%) ^c^ |  |  |  |  |  |  |  | 283 (19.7) | 459 (31.9) | 92 (6.4) | 387 (26.9) | 218 (15.1) | 1439 (100) |  |  |  |  |
|  | Avoiding travel | Yes |  |  |  |  |  |  |  | 221 | 350 | 69 | 290 | 162 | 1092 (76.2) | 69.46* | 69.86* | 27.45* | 0.42 |
|  |  | No |  |  |  |  |  |  |  | 60 | 107 | 23 | 97 | 55 | 342 (23.8) |  |  |  |  |
|  |  | Total (%) ^c^ |  |  |  |  |  |  |  | 281 (19.6) | 457 (31.9) | 92 (6.4) | 387 (27.0) | 217 (15.1) | 1434 (100) |  |  |  |  |

Notes.

Compliance at baseline in students responding to the 5-month follow-up (A) and to the 10-month follow-up (B).

Compliance at 5 months post-baseline in students responding to the 10-month follow-up (C).

Self-reported change in academic self-efficacy at the 5-month follow-up (a & b) and at the 10-month follow-up (c).

df = degrees of freedom.

*p < .05
