# Supplementary material for: Non-compliance with COVID-19 Health Recommendations: Five- and Ten-Month Effects on Mental Health and Academic Self-efficacy Among University Students in Sweden
Source: Int J Behav Med. 2024 Dec 30;33(3):452–60. doi: 10.1007/s12529-024-10343-w (PMC13342285; doi:10.1007/s12529-024-10343-w)
Supplement: Supplementary file 4 — Supplementary file4 (DOCX 17 KB) [file 12529_2024_10343_MOESM4_ESM.docx]

**Online Supplementary Table 3.** Non-compliance with Covid 19 public health recommendations at baseline and at 5-month follow-up in relation to self-reported change in mental health at 5- and 10-month follow-ups. Medians of the marginal posterior distributions of odds ratios with 2.5% and 97.5% percentiles are reported, followed by the posterior probability that the odds ratio is greater or less than 1 (in the direction of the median).

|  | | Self-reported change in mental health | | | | | |
| --- | --- | --- | --- | --- | --- | --- | --- |
|  |  | 5-month follow-up | | | 10-month follow-up | | |
|  |  | Worse vs. No change | Better vs. No change | Both vs. No change | Worse vs. No change | Better vs. No change | Both vs. No Change |
| Compliance with Covid-19 public health recommendations at baseline | Not staying at home  vs. Compliant | 0.52 (0.35; 0.77)/>99.9% | 0.96 (0.53; 1.68)/55.9% | 0.49 (0.31; 0.74)/>99.9% | 0.75 (0.5; 1.14)/91.3% | 0.77 (0.38; 1.49)/77.8% | 0.52 (0.32; 0.82)/99.8% |
|  | Not keeping a distance  vs Compliant | 0.67 (0.43; 1.04)/96.4% | 0.99 (0.49; 1.9)/51.6% | 0,89 (0.54; 1.43)/68.7% | 0.74 (0.45; 1.22)/88.3% | 0.52 (0.21; 1.19)/93.9% | 0.91 (0.54; 1.57)/62.7% |
|  | Not avoiding risk groups  vs Compliant | 0.36 (0.17; 0.73)/99.8% | 0.26 (0.07; 0.82)/99.1% | 0.67 (0.31; 1.41)/85.1% | 0.61 (0.27; 1.35)/89.0% | 0.12 (0.02; 0.61)/99.5% | 0.88 (0.37; 2.06)/61.6% |
|  | Not avoiding transportation  vs Compliant | 0.98 (0.71; 1.35)/54.9% | 0.96 (0.57; 1.59)/56.2% | 0.95 (0.67; 1.34)/62.0% | 1.24 (0.88; 1.77)/88.6% | 0.76 (0.42; 1.35)/81.9% | 0.85 (0.58; 1.26)/79.0% |
|  | Not avoiding travel  vs Compliant | 0.87 (0.57; 1.33)/73.1% | 1.02 (0.52; 1.92)/52.2% | 0.76 (0.47; 1.22)/87.0% | 1.20 (0.74; 1.97)/77.5% | 0.74 (0.3; 1.66)/76.5% | 1.25 (0.74; 2.13)/79.4% |
| Compliance with Covid-19 public health recommendations at 5 months post-baseline | Not staying at home  vs. Compliant |  |  |  | 0.95 (0.70; 1.29)/62.0% | 0.57 (0.33; 0.98)/97.9% | 0.75 (0.54; 1.04)/95.6% |
|  | Not keeping a distance  vs Compliant |  |  |  | 1.02 (0.69; 1.52)/54.3% | 0.83 (0.4; 1.63)/70.5% | 1.10 (0.72; 1.68)/66.9% |
|  | Not avoiding risk groups  vs Compliant |  |  |  | 0.84 (0.54; 1.32)/78.4% | 0.53 (0.21; 1.2)/93.5% | 1.15 (0.72; 1.84)/72.5% |
|  | Not avoiding transportation  vs Compliant |  |  |  | 1.32 (0.96; 1.81)/95.7% | 1.17 (0.69; 1.98)/72.4% | 1.19 (0.84; 1.67)/83.9% |
|  | Not avoiding travel  vs Compliant |  |  |  | 0.94 (0.67; 1.32)/64.8% | 0.87 (0.48; 1.54)/68.4% | 0.79 (0.55; 1.16)/88.7% |
