# Supplementary material for: Non-compliance with COVID-19 Health Recommendations: Five- and Ten-Month Effects on Mental Health and Academic Self-efficacy Among University Students in Sweden
Source: Int J Behav Med. 2024 Dec 30;33(3):452–60. doi: 10.1007/s12529-024-10343-w (PMC13342285; doi:10.1007/s12529-024-10343-w)
Supplement: Supplementary file 5 — Supplementary file5 (DOCX 17 KB) [file 12529_2024_10343_MOESM5_ESM.docx]

**Online Supplementary Table 4.** Non-compliance with Covid 19 public health recommendations at baseline and at 5-month follow-up in relation to self-reported change in academic self-efficacy at 5- and 10-month follow-ups. Medians of the marginal posterior distributions of odds ratios with 2.5% and 97.5% percentiles are reported, followed by the posterior probability that the odds ratio is greater or less than 1 (in the direction of the median).

|  | | Self-reported change in academic self-efficacy | | | | | | | |
| --- | --- | --- | --- | --- | --- | --- | --- | --- | --- |
|  |  | 5-month follow-up | | | | 10-month follow-up | | | |
|  |  | Worse vs. No change | Better vs. No change | Both vs. No change | Not studying vs. No change | Worse vs. No change | Better vs. No change | Both vs. No Change | Not studying vs. No change |
| Compliance with Covid-19 public health recommendations at baseline | Not staying at home  vs. Compliant | 0.74 (0.46; 1.19)/89.6% | 0.69 (0.34; 1.34)/86.6% | 0.55 (0.34; 0.88)/99.4% | 0.63 (0.33; 1.14)/93.6% | 0.78 (0.47; 1.32)/82.1% | 1.11 (0.52; 2.31)/61.0% | 0.93 (0.55; 1.58)/61.2% | 1.12 (0.61; 2.02)/64.4% |
|  | Not keeping a distance  vs Compliant | 0.83 (0.48; 1.45)/74.2% | 1.03 (0.48; 2.15)/52.9% | 0.80 (0.46; 1.38)/78.7% | 0.77 (0.37; 1.55)/76.7% | 0.80 (0.43; 1.46)/76.4% | 1.24 (0.52; 2.83)/69.2% | 0.92 (0.5; 1.7)/60.0% | 0.59 (0.27; 1.26)/91.2% |
|  | Not avoiding risk groups  vs Compliant | 0.43 (0.18; 1.01)/97.4% | 0.62 (0.19; 1.85)/80.4% | 1.04 (0.47; 2.34)/54.1% | 0.34 (0.11; 1.00)/97.5% | 0.34 (0.12; 0.89)/98.5% | 0.56 (0.13; 2.01)/81.0% | 0.88 (0.35; 2.21)/60.8% | 0.23 (0.06; 0.79)/99.1% |
|  | Not avoiding transportation  vs Compliant | 1.39 (0.93; 2.09)/94.5% | 0.71 (0.39; 1.27)/87.5% | 1.11 (0.74; 1.67)/69.5% | 1.14 (0.67; 1.92)/67.4% | 1.32 (0.85; 2.05)/89.5% | 0.83 (0.43; 1.57)/72.2% | 1.17 (0.75; 1.82)/75.3% | 1.69 (1.02; 2.82)/97.9% |
|  | Not avoiding travel  vs Compliant | 1.27 (0.75; 2.18)/81.2% | 0.82 (0.36; 1.74)/69.3% | 1.04 (0.61; 1.79)/55.9% | 1.21 (0.60; 2.41)/71.0% | 1.51 (0.83; 2.74)/91.4% | 1.22 (0.5; 2.86)/67.9% | 1.32 (0.72; 2.43)/81.5% | 1.23 (0.6; 2.51)/71.4% |
| Compliance with Covid-19 public health recommendations at 5 months post-baseline | Not staying at home  vs. Compliant |  |  |  |  | 1.11 (0.62; 2.00)/64.1% | 0.48 (0.20; 1.09)/96.0% | 0.76 (0.42; 1.36)/82.2% | 1.34 (0.70; 2.58)/81.2% |
|  | Not keeping a distance  vs Compliant |  |  |  |  | 1.18 (0.57; 2.44)/67.2% | 1.41 (0.50; 3.82)/74.6% | 0.97 (0.46; 2.02)/53.9% | 0.87 (0.37; 1.98)/63.1% |
|  | Not avoiding risk groups  vs Compliant |  |  |  |  | 1.16 (0.70; 1.91)/71.7% | 1.26 (0.59; 2.59)/73.0% | 0.95 (0.57; 1.58)/58.0% | 1.36 (0.79; 2.33)/87.0% |
|  | Not avoiding transportation  vs Compliant |  |  |  |  | 1.34 (0.95; 1.89)/95.3% | 0.78 (0.44; 1.34)/81.4% | 1.20 (0.85; 1.7)/84.7% | 1.47 (0.99; 2.17)/97.2% |
|  | Not avoiding travel  vs Compliant |  |  |  |  | 1.03 (0.70; 1.51)/55.6% | 1.15 (0.62; 2.07)/67.5% | 1.22 (0.84; 1.79)/85.2% | 1.19 (0.77; 1.89)/78.7 |
